# Fusion-Bloom: fusion detection in assembled transcriptomes

**DOI:** 10.1093/bioinformatics/btz902

**Published:** 2019-12-02

**Authors:** Readman Chiu, Ka Ming Nip, Inanc Birol

**Affiliations:** 1 Canada’s Michael Smith Genome Sciences Centre, BC Cancer, Vancouver, BC V5Z 4S6, Canada; 2 Bioinformatics Graduate Program, University of British Columbia, Vancouver, BC V6H 3N1, Canada; 3 Department of Medical Genetics, University of British Columbia, Vancouver, BC V6H 3N1, Canada

## Abstract

**Summary:**

Presence or absence of gene fusions is one of the most important diagnostic markers in many cancer types. Consequently, fusion detection methods using various genomics data types, such as RNA sequencing (RNA-seq) are valuable tools for research and clinical applications. While information-rich RNA-seq data have proven to be instrumental in discovery of a number of hallmark fusion events, bioinformatics tools to detect fusions still have room for improvement. Here, we present Fusion-Bloom, a fusion detection method that leverages recent developments in *de novo* transcriptome assembly and assembly-based structural variant calling technologies (RNA-Bloom and PAVFinder, respectively). We benchmarked Fusion-Bloom against the performance of five other state-of-the-art fusion detection tools using multiple datasets. Overall, we observed Fusion-Bloom to display a good balance between detection sensitivity and specificity. We expect the tool to find applications in translational research and clinical genomics pipelines.

**Availability and implementation:**

Fusion-Bloom is implemented as a UNIX Make utility, available at https://github.com/bcgsc/pavfinder and released under a Creative Commons License (Attribution 4.0 International), as described at http://creativecommons.org/licenses/by/4.0/.

**Supplementary information:**

Supplementary data are available at *Bioinformatics* online.

## 1 Introduction

Gene fusions have long been known as drivers for both development and progression in various tumour. Over the years, a number of software tools have been developed to detect gene fusions from RNA sequencing (RNA-seq) data (Kumar *et al.*, 2016). The algorithms of many of these tools typically involve detection of clusters of split single-read and discordant read-pair alignments against the reference genome or transcriptome. Alternatively, other tools use *de novo* assembly methods to produce sequences longer than the raw reads for more accurate sequence mapping before fusion detection. Although developments in long read technologies may alter this assessment in the future, the current cost-benefit-value balance still favours short reads for many applications.

Here, we describe a pipeline called Fusion-Bloom, which combines the use of a new *de novo* transcriptome assembler, RNA-Bloom (Nip *et al.*, 2019) and a versatile assembly-based structural variant caller, PAVFinder (Chiu *et al.*, 2018), for fusion detection. We demonstrate the performance of Fusion-Bloom on simulated and experimental RNA-seq datasets. We benchmarked its estimation accuracy and computational resource requirements in comparison to those of six RNA-seq fusion detection tools.

## 2 Materials and methods

Fusion-Bloom is implemented as a UNIX Make utility, which automates three analysis stages: assembly, alignment and analysis (Supplementary Fig. S1). In the first stage, paired RNA-seq reads are assembled by RNA-Bloom with the option ‘-chimera–extend–stratum 01’ to improve its reconstruction of full-length chimeric transcripts in low abundance. To expedite processing, Fusion-Bloom only retains RNA-Bloom contigs longer than the first quartile length of the entire assembly for downstream analysis. Contigs are then aligned against both the reference genome and annotated transcripts. Reference transcript alignment provides a computationally inexpensive yet useful complement to the genome alignment for chimera identification. Raw RNA-seq reads are also aligned to the contigs for: (i) filtering of mis-assembled chimeric junctions and (ii) estimating the expression levels of putative fusions. Based on these alignments, PAVFinder detects potential fusions and reports its results in BEDPE format (Supplementary Table S1).

## 3 Results

**Fig. 1.**
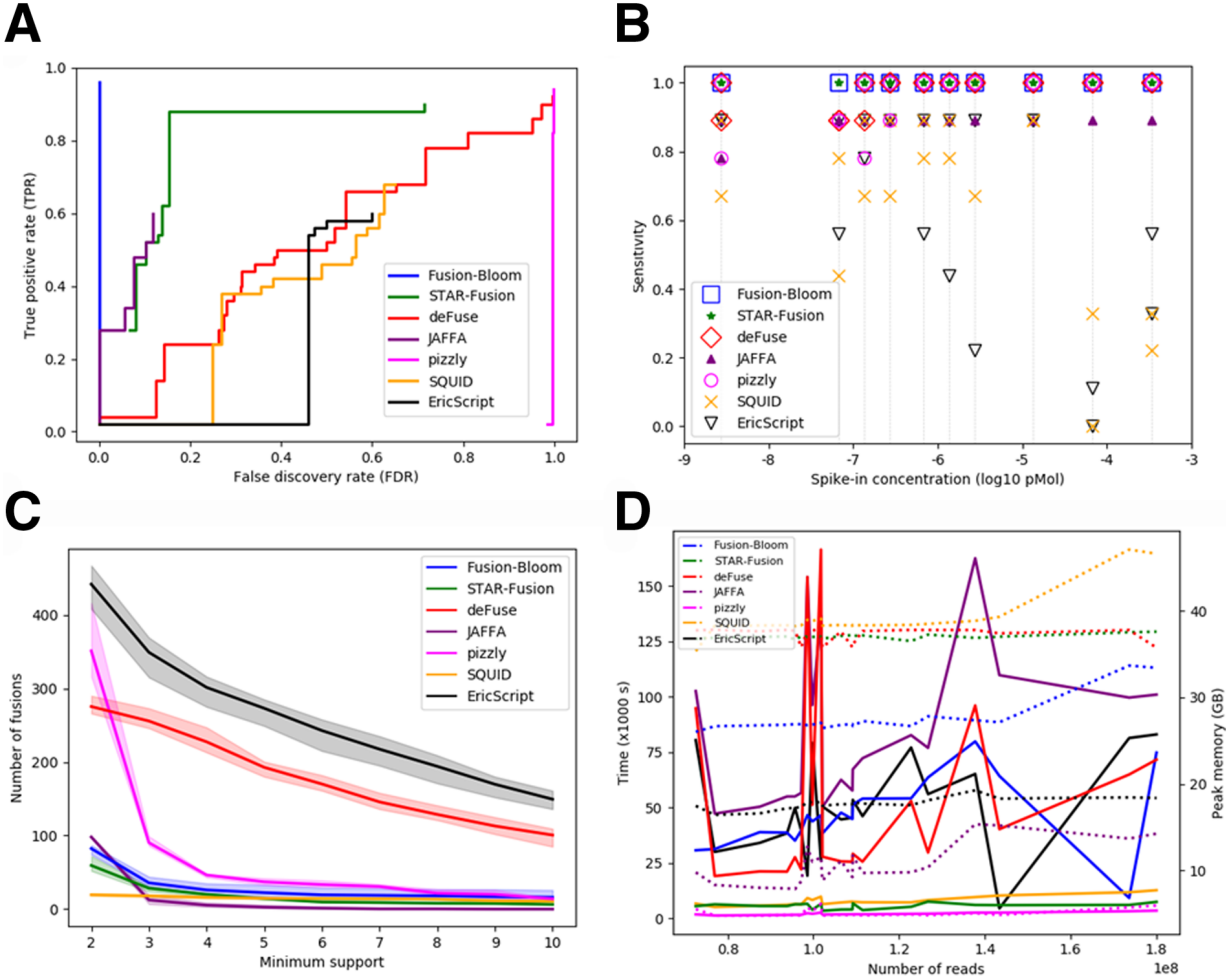
Benchmarking results of Fusion-Bloom and six other fusion detection tools. (**A**) Sensitivity-versus-precision plot on simulated FusionMap fusions combined with simulated reads representing reference transcripts in similar total abundance. (**B**) Sensitivity benchmark using 10 replicates with 9 fusions spiked in at different molarities (grey lines). (**C**) Total number of fusions reported in healthy blood samples in relation to minimum level of read support. (**D**) Wall-clock time (left Y-axis, solid lines) and peak memory usage (right Y-axis, dotted lines) benchmarked on spike-in samples. All the tools were run using 12 threads on a single Intel Xeon E5-2699 v3 2.30 GHz 36-core machine running CentOS 6

We took a commonly-used benchmarking dataset consisting of 50 fusions to compare the performance of Fusion-Bloom against 5 other fusion detection tools: deFuse (McPherson *et al.*, 2011), STAR-Fusion (Haas *et al.*, 2017), JAFFA (Davidson *et al.*, 2015), pizzly (Melsted *et al.*, 2017), SQUID (Ma *et al.*, 2018) and EricScript (Benelli *et al.*, 2012) (Supplementary Table S2). Fusion-Bloom out-performed other tools by detecting the largest number of fusions (48) with zero false-positive (Supplementary Fig. S2). To better mimic data from tumour transcriptomes, we repeated the benchmarking experiment by combining the fusion-only dataset with an additional dataset comprising similar number of reads simulated from the ENSEMBLE annotation. We generated sensitivity-versus-precision plots of the tools (Fig. 1A) by filtering reported events with different read support levels represented by breakpoint-spanning reads and flanking read pairs (Supplementary Table S3). Fusion-Bloom was the best performer in this test; it does not produce any false-positives within the entire range of support levels (hence a vertical line). At the other end of the spectrum, pizzly’s false positives remained high at all minimum support levels evaluated and thus produced a consistently high false discovery rate (FDR). The other tools displayed a more gradual linear relationship between true positive rate and FDR in response to the range of minimum support levels tested.

A publicly available dataset consisting of synthetic fusion transcripts spiked in at a wide range of molarity levels to total RNA provides another useful benchmarking test of sensitivity (Tembe *et al.*, 2014). The dataset is composed of 20 samples, each harbouring 9 fusions spiked in at 10 different molarities to total RNA in duplicate. Fusion-Bloom and STAR-Fusion were the most sensitive tools as they were capable of detecting all fusions at all molarities in both replicates (Fig. 1B, Supplementary Table S4).

To assess the tools’ specificity in experimental data, we analyzed three RNA-seq samples that are technical replicates of a whole-blood sample pooled from five healthy donors (Zhao *et al.*, 2015). While we cannot assume all fusions detected in healthy individuals are false positives without validation, we expect the majority of reported events are likely false-positives. We made a plot of the total number of fusions at different levels of minimum support to determine an optimal cutoff for comparison (Fig. 1C). Using a minimum of 4 spanning reads as the cutoff, JAFFA consistently reports the fewest number of fusions (5), whereas EricScript (301) and deFuse (227) report the most. SQUID, STAR-Fusion, Fusion-Bloom and pizzly report an average number of 16, 20, 26 and 46 fusions, respectively.

We benchmarked the computing performance of the tools using the 20 spike-in samples which contained 73 to 180 million read pairs (Fig. 1D). On average, Fusion-Bloom requires 10–12 h to process one hundred million read pairs. Although this is slower than alignment-based methods such as pizzly and STAR-Fusion, we think that *de novo* assembly is a valuable approach in that it provides base-pair precision of fusion breakpoints, and can also be used for detecting other long-range transcriptome rearrangement such as tandem-duplications and splice variants (Chiu *et al.*, 2018).

## Funding

This work was supported by Genome Canada and Genome BC [281ANV]; and the National Institutes of Health [R01HG007182]. Scholarship funding was provided by the Natural Sciences and Engineering Research Council of Canada. The content is solely the responsibility of the authors and does not necessarily represent the official views of the funding organizations.

*Conflict of Interest*: none declared.

## Supplementary Material

btz902_Supplementary_Data

## References

[btz902-B1] Benelli M. et al (2012) Discovering chimeric transcripts in paired-end RNA-seq data by using EricScript. Bioinformatics, 28, 3232–3239. 23093608 10.1093/bioinformatics/bts617

[btz902-B2] Chiu R. et al (2018) TAP: a targeted clinical genomics pipeline for detecting transcript variants using RNA-seq data. BMC Med. Genom., 11, 79. 10.1186/s12920-018-0402-6 PMC6131862 30200994

[btz902-B3] Davidson N.M. et al (2015) JAFFA: high sensitivity transcriptome-focused fusion gene detection. Genome Med., 7, 43. 26019724 10.1186/s13073-015-0167-x PMC4445815

[btz902-B4] Haas B. et al (2017) STAR-fusion: fast and accurate fusion transcript detection from RNA-Seq. *bioRxiv*, 120295.

[btz902-B5] Kumar S. et al (2016) Comparative assessment of methods for the fusion transcripts detection from RNA-Seq data. Sci. Rep., 6, 21597. 26862001 10.1038/srep21597 PMC4748267

[btz902-B6] Ma C. et al (2018) SQUID: transcriptomic structural variation detection from RNA-seq. Genome Biol., 19, 52. 29650026 10.1186/s13059-018-1421-5 PMC5896115

[btz902-B7] McPherson A. et al (2011) deFuse: an algorithm for gene fusion discovery in tumor RNA-Seq data. PLoS Comput. Biol., 7, e1001138. 21625565 10.1371/journal.pcbi.1001138 PMC3098195

[btz902-B8] Melsted P. et al (2017) Fusion detection and quantification by pseudoalignment. *bioRxiv*, 166322.

[btz902-B9] Nip K.M. et al (2019) RNA-bloom provides lightweight reference-free transcriptome assembly for single cells. bioRxiv, 701607.

[btz902-B10] Tembe W.D. et al (2014) Open-access synthetic spike-in mRNA-seq data for cancer gene fusions. BMC Genomics, 15, 824. 25266161 10.1186/1471-2164-15-824 PMC4190330

[btz902-B11] Zhao S. et al (2015) Comparison of stranded and non-stranded RNA-seq transcriptome profiling and investigation of gene overlap. BMC Genomics, 16, 675. 26334759 10.1186/s12864-015-1876-7 PMC4559181

