# Supplementary material for: Fusion-Bloom: fusion detection in assembled transcriptomes
Source: Bioinformatics. 2019 Dec 2;36(7):2256–7. doi: 10.1093/bioinformatics/btz902 (PMC7141844; doi:10.1093/bioinformatics/btz902)
Supplement: btz902_Supplementary_Data [file btz902_supplementary_data.doc]

# Fusion-Bloom: fusion detection in assembled transcriptomes

**Supplemental Materials**

Fig. S1. Fusion-Bloom pipeline

Fig. S2. Comparison of true and false positives in fusion calls from different tools on simulated dataset of solely 50 fusions

Table S1. PAVFinder output description

Table S2. Fusion detection tools and command lines used in study

Table S3. Output fields of different tools used to collect read support levels for generating sensitivity-vs-precision plot (Fig 1A)

Table S4. Sensitivities of different tools in spike-in dataset


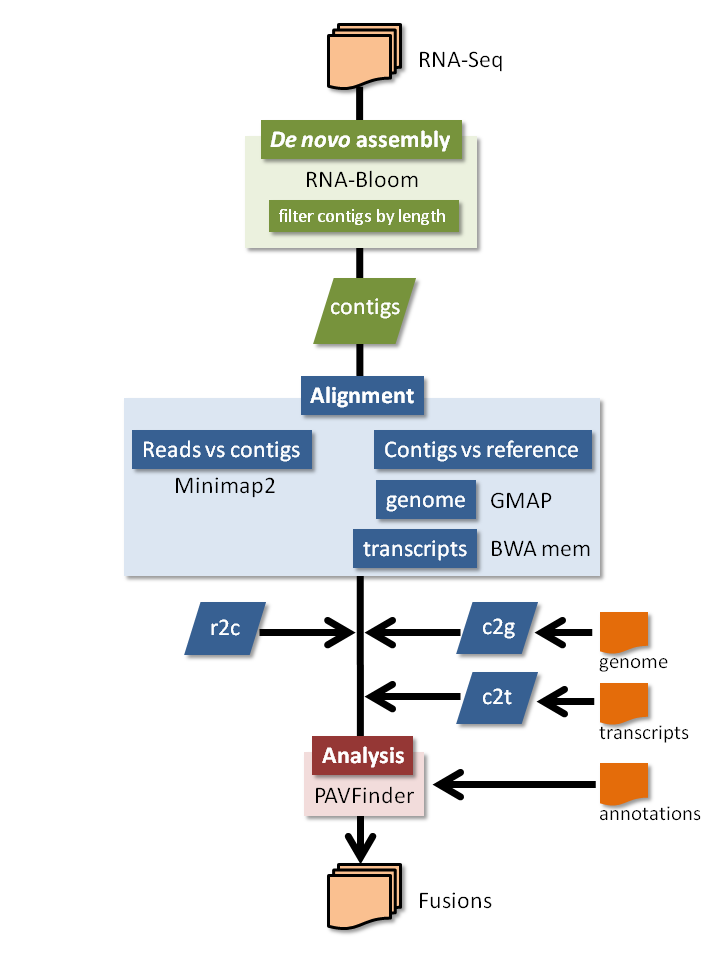


**Fig. S1. Fusion-Bloom pipeline.** Versions of software used: RNA-Bloom (v1.2), GMAP (2014-12-28), BWA mem (0.7.12), Minimap2 (2.17-r941), and PAVFinder (v1.6). Reference sequence and annotation files for analysis in this study: human genome sequence (hg38 UCSC), human gene annotation (GENCODE v26). Reference transcript sequences were generated by the utility script “extract_transcript_sequence.py” provided in PAVFinder using the annotation file in GTF format. Contig length filtering is executed internally within Fusion-Bloom by calling a custom AWK script called “filter_fasta” also provided under PAVFinder.


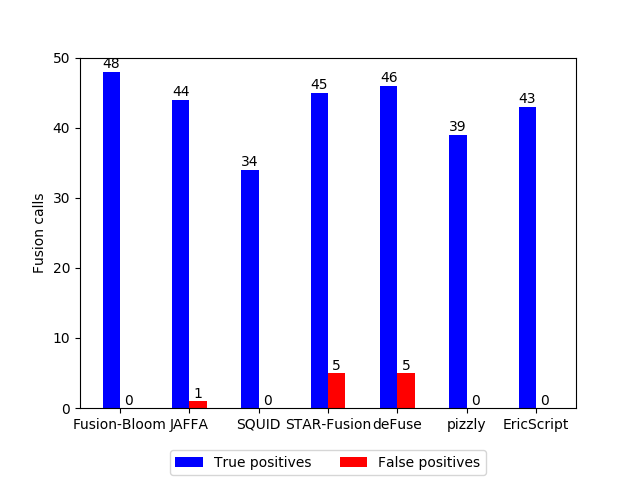


**Fig. S2. Comparison of true and false positives in fusion calls from different tools on simulated dataset of solely 50 fusions.** True and false positives were labeled based only on matching partner genes in fusions (with aliases taken into account). Multiple calls on the same two genes were counted as distinct calls, i.e. one of the calls was counted as true positive while the additional one(s) as false positive(s).

| **Field** | **Description** |
| --- | --- |
| chrom1 | chromosome of gene1 |
| start1 | breakpoint coordinate (minus 1) of gene1 |
| end1 | breakpoint coordinate of gene1 |
| chrom2 | chromosome of gene2 |
| start2 | breakpoint coordinate of gene2 |
| end2 | breakpoint coordinate (plus 1) of gene1 |
| name | arbitrary identification number of event |
| score | "." for fulfilling bedpe format |
| strand1 | strand of gene1 (always "+" regardless of gene orientation) |
| strand2 | strand of gene2 (always "+" regardless of gene orientation) |
| orient1 | orientation of gene1 sequence relative to breakpoint ("L" for upstream, "R" for downstream) |
| orient2 | orientation of gene2 sequence relative to breakpoint ("L" for upstream, "R" for downstream) |
| event | event type: "fusion" or "read-through" |
| size | not applicable for fusion events |
| gene1 | symbol of gene1 (order based on contig sequence) |
| transcript1 | transcript ID of gene1 |
| transcript_break1 | transcript breakpoint coordinate of gene1 |
| exon1 | exon number of gene1 |
| exon_bound1 | whether gene1 breakpoint is on exon boundary (True/False) |
| gene2 | symbol of gene2 (order based on contig sequence) |
| transcript2 | transcript ID of gene2 |
| transcript_break2 | transcript breakpoint coordinate of gene2 |
| exon2 | exon number of gene2 |
| exon_bound2 | whether gene2 breakpoint is on exon boundary (True/False) |
| gene_5prime | symbol of 5' gene in fusion product |
| gene_3prime | symbol of 3' gene in fusion product |
| exon_5prime | exon number of 5' gene in fusion product |
| exon_3prime | exon number of 3' gene in fusion product |
| feature | gene features other than exons where the breakpoint lands, e.g. 5'UTR, 3'UTR |
| seq_id | contig identification number from assembly |
| seq_breaks | coordinates of breakpoint flanking bases inside contig(s) |
| ins_seq | not applicable for fusion events |
| homol_seq | microhomology sequence at breakpoint |
| homol_seq_coords | contig coordinates of microhomology sequence |
| novel_seq | non-template sequence at breakpoint |
| novel_seq_coords | contig coordinates of non-template sequence |
| copy_number_change | not applicable for fusion events |
| repeat_seq | not applicable for fusion events |
| in_frame | whether fusion product is in-frame or not (True/False) |
| splice_motif | not applicable for fusion events |
| probe | short breakpoint-spanning sequence extracted from contig |
| support_span | breakpoint-spanning contig coordinates used for identifying spanning reads |
| spanning_reads | number of reads spanning breakpoint with at least N (default: 4) bases |
| flanking_pairs | number of read pairs flanking but not overlapping breakpoint |

**Table S1. PAVFinder output description.** Explanation of PAVFinder fusion output.

| **Tool** | **Version** | **Command** |
| --- | --- | --- |
| Fusion-Bloom | 1.6 | fusion-bloom profile=fusion-bloom.profile left=<reads_1.fastq.gz> right=<reads_2.fastq.gz> readlen=<read_len> outdir=<outdir> |
| JAFFA | 1.0.9 | bpipe run -n 12 -p annotation=genCode26 JAFFA_direct.groovy <reads_1.fastq.gz> <reads_2.fastq.gz> |
| STAR-Fusion | 1.1.0 | STAR-Fusion --genome_lib_dir GRCh38_gencode_v26_CTAT_lib_Nov012017/ctat_genome_lib_build_dir//ref_genome.fa.star.idx --left_fq <reads_1.fastq.gz> --right_fq <reads_2.fastq.gz> --output_dir <outdir> --CPU 12 |
| deFuse | 0.8.1 | defuse_run.pl -c <defuse_config.txt> -d defuse_ref -1 <reads_1.fastq.gz> -2 <reads_2.fastq.gz> -o <outdir> -p 12 |
| pizzly | 0.43.1 | kallisto quant -t 12 -i index.idx --fusion -o <outdir> <reads_1.fastq.gz> <reads_2.fastq.gz> |
|  | 0.37.3 | pizzly -k 31 --gtf gencode.v26.annotation.gtf.gz --fasta gencode.v26.transcripts.fixed.fa.gz --output <outdir> <fusion.txt> |
| SQUID | 2.5.3a | STAR --runThreadN 12 --genomeDir GRCh38_gencode_v26_CTAT_lib_Nov012017/ctat_genome_lib_build_dir//ref_genome.fa.star.idx --readFilesIn <reads_1.fastq.gz> <reads_1.fastq.gz> --readFilesCommand zcat --outFileNamePrefix <prefix> --outSAMtype BAM SortedByCoordinate --outReadsUnmapped Fastx --chimSegmentMin 20 --outSAMstrandField intronMotif |
|  | 1.5 | samtools view -Shb Chimeric.out.sam -o Chimeric.out.bam |
|  | 1.5 | squid -b Aligned.sortedByCoord.out.bam -c Chimeric.out.bam -G 1 -CO 1 -o squid1.5 |
| EricScript | 0.5.5b | ericscript.pl –db ericscript_db_homosapiens_ensembl84 –name <lib> --nthreads 12 –remove –o <oudtdir> <reads_1.fastq.gz> <reads_2.fastq.gz> |

**Table S2. Fusion detection tools and command lines used in study.**

| **Tool** | **Fields** |
| --- | --- |
| Fusion-Bloom | spanning_reads, flanking pairs |
| JAFFA | spanning reads, spanning_pairs |
| STAR-Fusion | JunctionReadCount, SpanningFragCount |
| deFuse | splitr_count, span_count |
| pizzly | paircount, splitcount |
| SQUID | score |
| EricScript | spanningreads, crossingreads |

**Table S3.** **Output fields of different tools used to collect read support levels for generating sensitivity-vs-precision plot (Fig 1A).** TPR (Y-axis) and FDR(X-axis) of all combinations of support values were calculated and the Pareto-front was shown for each tool.

TPR = # True fusions detected / # Simulated Fusions

FDR = # False fusions detected / # Detected Fusions

| **Sample** | **Molarity**  **(-log10 pMol)** | **EricScript** | **Fusion-Bloom** | **JAFFA** | **SQUID** | **STAR-Fusion** | **deFuse** | **pizzly** |
| --- | --- | --- | --- | --- | --- | --- | --- | --- |
| **SRR1659951** | 3.47 | 0.56 | 1.00 | 0.89 | 0.33 | 1.00 | 1.00 | 1.00 |
| **SRR1659960** | 3.47 | 0.33 | 1.00 | 0.89 | 0.22 | 1.00 | 1.00 | 1.00 |
| **SRR1659952** | 4.17 | 0.00 | 1.00 | 0.89 | 0.00 | 1.00 | 1.00 | 1.00 |
| **SRR1659961** | 4.17 | 0.11 | 1.00 | 0.89 | 0.33 | 1.00 | 1.00 | 1.00 |
| **SRR1659953** | 4.87 | 0.89 | 1.00 | 0.89 | 0.89 | 1.00 | 1.00 | 1.00 |
| **SRR1659962** | 4.87 | 0.89 | 1.00 | 0.89 | 0.89 | 1.00 | 1.00 | 1.00 |
| **SRR1659954** | 5.57 | 0.22 | 1.00 | 0.89 | 0.67 | 1.00 | 1.00 | 1.00 |
| **SRR1659963** | 5.57 | 0.89 | 1.00 | 0.89 | 1.00 | 1.00 | 1.00 | 1.00 |
| **SRR1659959** | 5.87 | 0.44 | 1.00 | 0.89 | 0.78 | 1.00 | 1.00 | 1.00 |
| **SRR1659968** | 5.87 | 0.89 | 1.00 | 0.89 | 0.89 | 1.00 | 1.00 | 1.00 |
| **SRR1659955** | 6.17 | 0.56 | 1.00 | 0.89 | 0.78 | 1.00 | 1.00 | 1.00 |
| **SRR1659964** | 6.17 | 0.89 | 1.00 | 0.89 | 0.89 | 1.00 | 1.00 | 1.00 |
| **SRR1659956** | 6.57 | 1.00 | 1.00 | 0.89 | 0.67 | 1.00 | 1.00 | 0.89 |
| **SRR1659965** | 6.57 | 0.89 | 1.00 | 0.89 | 0.89 | 1.00 | 1.00 | 1.00 |
| **SRR1659957** | 6.87 | 0.78 | 1.00 | 0.89 | 0.67 | 1.00 | 0.89 | 0.78 |
| **SRR1659966** | 6.87 | 0.89 | 1.00 | 0.89 | 0.89 | 1.00 | 1.00 | 1.00 |
| **SRR1659958** | 7.17 | 0.56 | 1.00 | 0.89 | 0.44 | 1.00 | 0.89 | 0.89 |
| **SRR1659967** | 7.17 | 0.89 | 1.00 | 0.89 | 0.78 | 1.00 | 0.89 | 0.89 |
| **SRR1544075** | 8.57 | 0.89 | 1.00 | 0.89 | 0.89 | 1.00 | 1.00 | 1.00 |
| **SRR1548811** | 8.57 | 0.89 | 1.00 | 0.78 | 0.67 | 1.00 | 0.89 | 0.78 |

Table S4. Sensitivities of different tools in spike-in dataset. Sensitivity is measured as the proportion of the nine spike-in fusions detected.

References

Li, H. (2013) Aligning sequence reads, clone sequences and assembly contigs with BWA-MEM, *arXiv*, 1303.3997.

Li, H. (2018) Minimap2: pairwise alignment for nucleotide sequences, *Bioinformatics*, **34**, 3094-3100.

Wu, T.D. and Watanabe, C.K. (2005) GMAP: a genomic mapping and alignment program for mRNA and EST sequences, *Bioinformatics*, **21**, 1859-1875.
